# “I just want to move": preschool children’s perspectives on their physical literacy

**DOI:** 10.1186/s12889-026-26403-7

**Published:** 2026-01-27

**Authors:** Lisa Borglund, Camilla Eriksson, Lena Almqvist, Maria Harder

**Affiliations:** 1 Department of Public Health Sciences, Mälardalen University, Västerås, Sweden; 2 Department of Psychology, Mälardalen University, Västerås, Sweden; 3 Department of Caring Science, Mälardalen University, Västerås, Sweden

**Keywords:** Physical literacy, Preschool children, Public health, Qualitative study, Swedish preschool

## Abstract

**Background:**

Movement is important for preschool-aged children’s development. Developing physical literacy at an early age can benefit children’s health and their relationship with movement and physical activity in the long term. This study aimed to explore preschool children’s perspectives on their physical literacy, focusing on its core components: knowledge and understanding, motivation, self-confidence, and physical competence.

**Methods:**

A qualitative study was conducted at six Swedish preschools, with 25 preschool children aged five to six years as participants. Semi-structured interviews were carried out, and the interviews were audio- and video-recorded, transcribed, and analysed through a deductive content analysis.

**Results:**

Children want to move and enjoy movement. They experience movement as connected to their feelings and they are motivated to move when it feels good and it is fun. Their motivation for movement also comes from inclusion in preschool decision-making, teachers’ participation in play, and opportunities to play with friends. The children express confidence in their movement skills, show awareness of their abilities, and display a variety of age-appropriate movements. They challenge themselves and draw inspiration from their peers in movement activities. Overall, the findings highlight the children’s awareness of their motivation, well-being, and abilities alongside the emotional aspects that make movement meaningful for them.

**Conclusions:**

This study contributes to the limited research on preschool children’s physical literacy by showing that children’s motivation for movement is connected to how movement makes them feel. Thus, from the children’s perspective, the affective domain of physical literacy is recognised as central to their engagement in movement. Including children’s perspective advances the understanding of physical literacy and underscores the importance of considering the children’s experiences when promoting physical literacy in both research and practice.

**Supplementary Information:**

The online version contains supplementary material available at 10.1186/s12889-026-26403-7.

## Background

Preschool-aged children enjoy moving, and movement plays an important role in their development [1–3]. Movement during the early years is associated with improved psychosocial health and overall quality of life [4, 5]. During the preschool years, children develop essential motor skills as well as self-confidence in their movement abilities, both of which are promoted by physical literacy [6–8]. Lack of movement and increased sedentary behaviour are growing public health concerns, even among the youngest population group [9]. This situation calls for an exploration of preschool children’s relationship with movement and how movement is promoted in this population.

The Swedish preschool has a crucial role in promoting children’s relationship with movement, as 87% of children aged one to six years, and 96% of all five-year-olds, attend preschool [10]. The municipalities in Sweden are responsible for ensuring access to preschool for all children in this age group, as part of the preschool’s inclusive and compensatory role to ensure all children have equal opportunities to learn and develop. Thus, the preschool’s work to counteract the disparities in children’s learning experiences, which can result from having a different language spoken at home, parents’ educational level, or the family’s socioeconomic status [11].

The Swedish preschool is part of the welfare system and is regulated by a national curriculum (Lpfö 18) [11], which applies to both private and municipality-owned preschools. With regard to children’s movement, the preschool curriculum is broad and open to interpretation. However, preschools must work to encourage children’s interest in physical activity, support the development of various movement skills, and promote knowledge of a healthy lifestyle. Play is central to achieving this, and the children need opportunities for teacher-led and free play throughout the day according to the curriculum [11]. Meanwhile, preschool teachers report a lack of agency and formal strategies for working with children’s movement and physical activity [12–14]. Furthermore, research indicates that children spend most of their preschool day being sedentary, doing routine activities such as eating, dressing, or waiting passively. Also, the children’s movement occurs primarily outdoors, with limited opportunities to move indoors, with the result that children are physically active for only a short period during their preschool day. Thus, preschools fall short of fulfilling their mandated mission, particularly regarding their health-promoting role [14–17].

An effective approach to promoting movement among preschool children is to encourage the development of physical literacy. Through physical literacy, children form a positive relationship with movement that is sustainable long-term [18]. However, encouraging a sustainable relationship with movement goes beyond simply telling children to move. Physical literacy is a multi-component theoretical framework containing individual attributes identified as four core components: knowledge and understanding, motivation, self-confidence, and physical competence, which are individual qualities needed to value and engage in movement throughout life. These core components occur within three domains: cognitive, affective, and physical, all of which must be considered to facilitate participation in movement activities [19].

Knowledge and understanding can be found within the cognitive domain. The domain involves identifying key factors that affect movement, knowing the health benefits of being active, and valuing safety measures. It also involves being aware of one’s abilities and limitations in different movements and various contexts. Motivation and self-confidence comprise the affective domain. These components relate to the individual’s enjoyment, enthusiasm, and belief in their movement abilities, which are crucial for making physical activity an integral and lasting part of life. The physical domain relates to the individual’s physical competencies, including the ability to develop and apply a range of movement competencies required to partake in various physical activities [19, 20].

The core components of physical literacy work interdependently and together influence the individual’s movement behaviour, forming a link between physical literacy and physical activity [21]. Physical literacy in children is associated with health indicators, such as better cardiorespiratory health, lower sedentary behaviour, and improved mental well-being. It can also aid the development of fundamental motor skills and lead to physically active children [6, 8, 22, 23]. By supporting the development of physical literacy early in life, children are more likely to build the motivation, confidence, and competence needed to sustain an active lifestyle across the lifespan and reduce the risk of lifestyle-related illness [19].

Early interventions promoting positive health behaviours, such as movement, have long-lasting effects on both mental and physical well-being [3]. Similarly, physical literacy interventions in preschool settings have been found to improve fundamental movement skills, boost children’s self-confidence in managing a variety of movements, and promote a positive attitude towards movement and physical activity [6, 23]. As the vast majority of young children attend preschool in Sweden, it is a pivotal setting for promoting physical literacy [24]. Yet it remains unclear how Swedish preschools promote children’s movement development and how the children perceive their relationship with movement at preschool.

Research on physical literacy in preschool-aged children, particularly within the Swedish context, is still limited. Most existing physical literacy research has relied on measurements and observations, with few qualitative approaches [25], especially involving preschool children’s own perspectives on their movement. This suggests that children’s perspectives of physical literacy may be overlooked. To understand children’s movement at preschool, there is a need to involve the children as active participants in research and thereby gain insight into how they perceive and experience their own physical literacy.

The aim of this study was to explore preschool children’s perspectives on their physical literacy, focusing on its core components: knowledge and understanding, motivation, self-confidence, and physical competence.

## Methods

This study employed a qualitative research design, using individual interviews for the data collection, and applied a deductive content analysis [26].

### Participants

In total, 82 preschool children were invited to participate in the study, and consent was provided by 27 children and their legal guardians. Written information about the study was distributed to legal guardians via the preschool teachers. Before the interviews began, the children were again verbally asked by the researcher if they wanted to participate, whereof two children withdrew their consent as they no longer wished to answer questions. Consequently, 25 preschool children (nine boys and 16 girls) participated in individual interviews. All children were aged five to six years and were able to express themselves verbally in Swedish. To the authors’ knowledge, none of the children had any developmental delays, physical impairments, or conditions requiring a diagnosis (e.g., ADHD or autism spectrum disorder). However, such information was not collected and therefore cannot be confirmed.

### Data collection

The interviews were conducted in six preschools, two privately owned and four municipality-owned, located in one medium-sized (50,000–200,000 residents) and two smaller (< 50,000 residents) municipalities in Sweden [27]. All interviews were carried out by the first author during the day at each respective preschool. They took place indoors in a separate room with only the first author and child present, to minimise interruptions from the other children and teachers. Four interviews started outdoors in the preschool yard, as the children were already playing outside. This arrangement was chosen to fit the preschool’s daily routines and to avoid disrupting the children’s schedule, and the interviews later continued indoors.

The interviews followed a semi-structured interview guide developed for this study (see supplementary file). The questions were based on the core components of physical literacy: knowledge and understanding, motivation, self-confidence, and physical competence [19]. Approximately five questions were asked for each core component (motivation, self-confidence, physical competence, knowledge and understanding), with follow up questions included when necessary.

The interviews were recorded by the first author using an audio recorder and a video camera. The video camera ensured that the child’s movements during the interview were captured. When interviewing young children, it is important to be flexible and adjust the interview situation to the child’s needs [28]. Therefore, each child chose where in the room they felt most comfortable, and the interviewer adjusted accordingly by sitting next to the child, on the floor, on chairs, or at a table, to help them feel relaxed. The children were also free to move around the room during the interview if they so wished. Interview length ranged from seven to 24 min (mean 12.5 min).

### Analysis

**Fig. 1:**
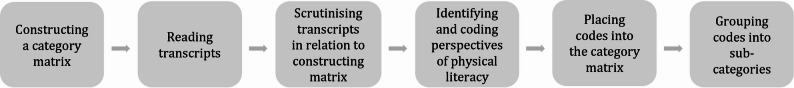
Outline of the analysis process

A deductive content analysis [26], guided by the physical literacy framework, to analyse the interviews, as shown in Fig. 1. The analysis was conducted using the qualitative data analysis software NVivo. A category matrix (Table 1) was constructed, with the core components of the physical literacy framework [19] serving as the main categories.

Firstly, each interview was transcribed verbatim by the first author. The video recordings were analysed to capture and describe the movement performed by each child during the interview. During transcription, all participants were de-identified and given a code (e.g., Participant 1). Thereafter, each transcript was read separately. Meaning units reflecting children’s perspectives on physical literacy were identified and scrutinised in relation to physical literacy as a theoretical concept and subsequently assigned to the appropriate predetermined category. The first author coded the meaning units based on the predetermined categories and the fundamental meaning of each unit. The last author double-checked and reviewed the coding, and any discrepancies were discussed between the first and last authors until consensus was reached. The first and last authors then jointly grouped the codes into subcategories based on similarities, while all authors discussed the process to ensure that no data were omitted. This step was done inductively, meaning that the subcategories were derived from the data itself, i.e., the children’s perspectives. The progression from deductive categorisation to inductive grouping of codes into subcategories further ensured that each code was placed within the most relevant subcategory.

## Results

The findings are based on the theoretical framework of physical literacy, and the main concepts are used as categories, namely: knowledge and understanding, motivation, self-confidence, and physical competence [19]. The children expressed their perspectives on their physical literacy through their feelings. How movement makes the children feel is how they experience, understand, and value movement, which will be depicted throughout the findings.

**Table 1:** Category matrix with the main categories and subcategories

Categories	Knowledge and understanding	Motivation	Self-confidence	Physical competence
Subcategories	To know and understand one’s body when moving To know and understand one’s body when being still	Taking part in desirable movement activities Taking part in undesirable movement activities	Believing in one’s own abilities Encountering Challenges Finding inspiration in others	Having movement skills in free play Taking part in organised play

### Knowledge and understanding

The category refers to the children’s knowledge of their bodies and their health in relation to movement and play. The children’s expressions of understanding are formed by how movement feels. Therefore, in the following, the children’s descriptions of being active show their feelings related to movement. The children also describe their knowledge of balancing between movement and rest, and the ability to listen to their body’s signals. They also describe their knowledge of how to utilise safety strategies to avoid being hurt when playing. Thus, expressing their understanding of their body and health whilst moving and being still. The category comprises two subcategories: *To know and understand one’s body when moving*, and *To know and understand one’s body when being still.*

#### To know and understand one’s body when moving

This subcategory describes the children’s knowledge of the health benefits of moving and their understanding of the benefits of listening to their body, as well as the children’s perspectives on how they feel when moving.

The children know and understand that it is good for their bodies to move. They say that their body wants to move and breathe. Furthermore, they express a feeling of happiness and contentment when they can play and run around freely. They also talk about their experience of happiness when moving, and say that certain movements, such as doing cartwheels, swinging high on a swing, or climbing high, are especially exciting. Thus, identifying the feeling they have when moving shows the essential qualities that influence their movement. The children’s understanding that they feel happy when the body is moving is shown in the following:

- I: How does it feel when you are rolling down the hill?
- P: [Sits straight in the chair, looks at the interviewer] “It feels like I’m a little meatball that shrinks when I roll.”
- I: Does it feel good?
- P: “Yes!”
- (Participant 11)

Furthermore, the children describe their knowledge and understanding of their bodies’ need to move. They highlight their understanding of the health benefits for one’s body when moving, as they say that it feels good to move outside in the preschool yard. Also, the children say that they can feel in their whole body that it is good to move. They specifically state that they feel it in their head, stomach, legs, and heart. They explicitly describe their understanding that movement makes the heart pump and that it is a good thing. Overall, they know and understand that it is good for their body and themselves to play, run, jump, and climb, which is shown in the following:

- I: How does it feel when you climb trees, play football, and run?
- P: [Sits at the table, drawing with a marker pen on a piece of paper, whilst looking at the interviewer] “Great. That I get to move.”
- I: Where in the body does it feel good?
- P: “In my heart because it beats more.”
- (Participant 8)

In addition to describing their knowledge and understanding of moving as something beneficial for the body, the children also voice their knowledge that eating food is good for their bodies. They express their knowledge that vegetables are important, but also that sometimes it can be good to eat a little bit of candy and ice cream.

The children also know, understand, and feel in their bodies that moving around and playing for a long time can result in tiredness and discomfort. They understand that it can sometimes be beneficial to be calm and let the body rest. The children reflect that running very fast or dancing, running, or spinning around for a long time can leave them feeling uncomfortable, tired, and sweaty, which causes a feeling of discomfort. Their bodies also hurt when moving a lot, which is expressed in “Sometimes my lungs hurt, I think it is because I run so much.”

The children also voice their understanding of valuing necessary safety measures when moving and playing. They say that hurting themselves when playing can make them uncomfortable, but that they develop strategies to avoid getting hurt again, and they are not deterred from playing because of it. Whilst expressing a feeling of unease when exerting themselves, the children are at the same time aware that it helps to rest and recover. They are excited to continue running, dancing, and playing after a short rest, despite having felt uncomfortable before. The children’s strategies are shown in the following:

- I: What do you think happens in the body when you play and move?
- P: “Sometimes when I run, it can hurt.”
- I: OK, how does that make you feel?
- P: “It feels OK, but sometimes I have to stop. And rest.”
- (Participant 24)

#### To know and understand one’s body when being still

This subcategory includes the children’s knowledge of their body whilst being still and their understanding that being still is not the same as being inactive. They also express their feelings of frustration about having to be still.

The children describe their knowledge of their bodies and their need to rest occasionally. Also, they understand their need for rest in relation to their prior activity of moving or playing. When the children describe their feelings of sitting down after playing, running, and dancing for some time, they say it is pleasant and that the body feels good when it can calm down: *The child turns the head and looks at the interviewer* “When I had been running, jumping, and spinning too much and I went to rest it felt better.” If the children are allowed to watch TV after playing, it makes them feel content, as well as when they get the opportunity to sit with a teacher and snuggle on the sofa, as this is relaxing and cosy.

However, the children also know that the act of being still is not necessarily the same as being inactive. They say that they do not wish to be completely still when sitting down. The children say that they play with toys and draw, and that they are also active during the morning assembly in preschool or warm-up in gymnastics. This activity is demonstrated by the fact that they are continuously playing with toys and moving around the room during the interviews. The children know their bodies well enough to understand that they desire to move. They describe feeling that their body needs to move, otherwise it can get angry and sad. The children sometimes feel uncomfortable being still if they are not able to play and be active at the same time. This is shown in the following:

- I: How does it feel when you have to sit still for a while?
- P: [Stands in the middle of the floor whilst spinning around in circles] “It is not fun.”
- I: How come?
- P: [Stops spinning, walks over to the table and leans against it] “Because I am not able to do anything then.”
- I: How does that make your body feel?
- P: [Looks at the interviewer whilst leaning against the table] “He gets sad. And angry.”
- I: Angry too?
- P: “Yes.”
- (Participant 5)

The children also voice their knowledge that being still and inactive is not good for their health, and it can make their hearts unwell: *The child draws with a marker pen on a piece of paper*,* looking down at the paper and says*, “Not so good. Then the heart stops beating and you die,” after reflecting on having to sit still. Being told to be still leads to a bad feeling that the children describe as restlessness and a lack of patience. They also find it tedious, and they express a feeling of frustration towards teachers who do not let them move around.

### Motivation

The children are motivated to move as they enjoy taking part in desirable movement activities with their friends, especially when they are involved in deciding what games to play. On the other hand, the children feel unmotivated to participate when they are left out of decisions, and when they have to do activities they find uninteresting. This is shown in the two subcategories: *Taking part in desirable movement activities* and *Taking part in undesirable movement activities*.

#### Taking part in desirable movement activities

This subcategory refers to the children’s perspectives on what motivates them to move and play outside and inside at preschool, highlighting their enthusiasm and interest in movement and play. The children feel that moving is fun and express their wish to move when they can choose their activities freely.

The children say that they are motivated to move. They think it is fun to move and play both inside and outside at preschool. Again, their descriptions convey that movement is connected to their emotions as they report that it feels fun and they enjoy moving in different settings, on their own, and with friends. The children share a variety of movement activities that they describe as the most fun to do, which include running, climbing, jumping, and dancing, among many things. The children express an interest in moving and playing, and they are motivated to move rather than being still. Their motivation and interest in movement are shown in the following:

- I: If you got to decide, would you rather move or be still?
- P: [Sits on a chair with one leg over the other, looking out the window] “Move!”
- I: Why would you choose that?
- P: [Puts both feet on the floor, starts dancing whilst sitting in the chair, moving the upper body] “Because I want to move a lot!”
- (Participant 14)

Furthermore, the children describe that playing with their friends is connected to their motivation to move. They want to create fun games with their friends, and they emphasise the importance of including everyone in choosing what games to play. It is also important to them that everyone feels included in the activities.

The children voice their desire for the teachers to involve them in deciding what they do in preschool. It makes them feel included when the preschool teachers allow them to choose activities, thus enhancing their motivation to participate in the activities. However, the children feel that the preschool teachers usually set rules and control what is not allowed, such as play fighting, running, and jumping indoors. The children express a willingness to adapt to the guidelines, as long as they feel included in decisions. By feeling heard and feeling involved, the children enjoy moving and being active in the activities. The children also voice their excitement about playing with the preschool teachers, and they prefer it if the teachers actively participate in the activities.

The children themselves differentiate between movement and play when they describe their motivation. Examples of movement activities they feel are fun are running very fast, jumping from rocks or other structures high up, climbing trees, and swinging on swings. They also enjoy dancing, spinning around, doing somersaults, and cartwheels. The children say that they are given more opportunities for movement when they are outside at preschool, as the inside premises can be restrictive in terms of space to move around. Therefore, they relate their favourite movement activities to being outside in the preschool yard, whilst their favourite activities indoors are related to playing. Indoor interactive games, such as playing house, using Lego, drawing, building puzzles, and using pearl plates are examples of activities they enjoy indoors. The children’s motivation to move and play is expressed by the following:

- I: What do you think is the most fun thing to do at preschool?
- P: [Stands up from the sofa and starts running around the room] “To run around. I’m superfast at running!”
- (Participant 12)

####  Taking part in undesirable movement activities

This subcategory includes the children’s perspectives on not being involved in what happens at preschool, as well as their perspectives on having to take part in undesirable activities. The children express a lack of motivation when not having the opportunity to be involved in decisions, making them feel uneasy. When they need to take part in activities that they are uninterested in, they feel bored. This indicates a difference in what affects their motivation, contrasting how they feel when excluded or when bored.

The lack of involvement in the decision process of choosing activities relates to the children feeling unprepared for what will happen. They say that both the preschool teachers and some of the other children decide over their heads at times. This leaves them feeling that their voice does not matter and that they do not want to participate in the activities. When their friends dictate what they will do, it creates a feeling of unease, which is shown in the following:

- I: Are there any movements you find boring?
- P: [Sits on a low table, crosses their legs, and looks around the room] “Mostly I like to play all the games that the others choose, but sometimes I like to play what I want. Then they say they don’t want to play with me.”
- (Participant 7)

The children also share activities at preschool that they find boring, and that make them uninterested in participating. They feel unmotivated to clean up after playing, when listening to the teachers at morning assembly, and playing games, which they find dull. The children express a desire to be able to choose what to do and find it unrewarding to have to comply with the rules set by the preschool teachers.

### Self-confidence

The children’s self-confidence in their movement skills demonstrates their self-assurance, as they believe in their own skills, but also shows their enthusiasm and enjoyment in taking inspiration from others and encountering challenges. The category comprises three subcategories: *Believing in one’s own abilities*,* Encountering challenges*,* and Finding inspiration in others.*

#### Believing in one’s own abilities

This subcategory highlights the children’s self-confidence in their movements, both movements they can and want to do. Their belief in their own abilities seems to be connected to how easy and enjoyable they perceive the movements. Their self-confidence is also related to the support the children receive from people around them.

The children express their self-confidence in the movements they feel they are best at, which include, for example, dancing, climbing, and running. They say that their ability to do certain movements is connected to how easy and fun they are to do. The children express a belief in their abilities because people around them, for example, their parents, grandparents, and teachers, have complimented them on their movement ability. The children’s self-confidence in certain movements, which they find fun, is shown in the following:

- I: What movement do you think you are best at?
- P: “I’m best at doing somersaults!” [Stands up on the floor, squats down whilst placing both hands on the floor, pushes their head towards their chest, and curves their back. Then stands back up straight again.]
- I: Oh! Why do you think you’re best at that?
- P: “Because it’s so much fun!”
- I: Is it difficult?
- P: “Noooooo!”
- (Participant 2)

The children mention various movement skills that they feel they are best at because they have done the movement many times before, and it is a part of their everyday life. For example, climbing in trees and on rocks, which they do both at preschool and at home, hence they feel they are very good at it. The children’s movement abilities are not always associated with one particular movement skill, as they express self-confidence and ability in doing many, if not all, movements they know and can perform: *The child sits on the bench next to the interviewer and looks up at the interviewer and says*: “I can do everything I know!”

#### Encountering challenges

This subcategory presents movement challenges the children have encountered and their strategies for conquering them. They also describe movement skills that are perceived as too difficult or scary, as well as skills they desire to have and want to try, but feel unable to attempt, as they are too young.

The children say that they dare to try new movements and want to keep trying to master new skills. They voice a sense of pride when they describe challenges they have encountered and mastered, thus achieving new movement skills. The children’s self-confidence in trying and mastering new skills is shown in the following:

- I: Do you dare try new things?
- P: Sits on a bench opposite the interviewer and nods their head.
- I: You mentioned you try to walk when you do a handstand. How can you dare try to do that?
- P: [Looks up at the interviewer] “I just lean back and think it will work.”
- I: So, you just try a little?
- P: “Yes.”
- (Participant 23)

However, although the children express their self-confidence in movement, they also voice their doubts about their abilities to perform specific movements. Movements such as doing cartwheels, backflips, skiing, and hand walking are perceived as difficult and therefore challenging. Although the children express a desire to overcome the challenges and master the skills, they also say that some movements seem too difficult, making them feel insecure and scared. This can make them refrain from trying, or if they have tried, stop them from attempting it again. This insecurity is related to the children being unsure of what the movement will involve or worrying that they may get hurt.

Furthermore, when the children encounter challenges, they voice their insecurities, for example, when a movement looks complicated or if the setting is unsuitable, such as doing somersaults on a hard surface, rather than on a soft mattress. The children are also aware that certain challenges may be easier to handle when they are a bit older, implying their awareness that their self-confidence in trying new movements may develop over time, which is shown in the following:

- I: Do you want to try new things?
- P: “No, not so much.”
- I: Why don’t you want to try new things?
- P: [Gets up from the floor, walks over to a balancing plate, and starts balancing] “Because I’m scared. I’m not eight years old. I’m only five. My sister is eight.”
- (Participant 20)

#### Finding inspiration in others

This subcategory includes where the children look for inspiration to learn new skills, and how they compare their abilities with their friends at preschool, thus highlighting their awareness of their self-confidence in movement skills.

The children say that practice is key to strengthening their self-confidence in mastering new movement skills, indicating their willingness to acquire more abilities. They look to friends, older siblings, and parents for support as well as inspiration for this. The children also find inspiration when seeing people do movements, they wish to learn themselves, for example, on; they wish to learn themselves, for example, from TV or watching older children in their neighbourhood. When the children see movements they cannot do themselves, they believe that they can also acquire that skill if they practice. The children also compare their own abilities with those of their peers at preschool. On occasion, they identify that their friends have specific competences that they wish to acquire themselves, for example, building a really good fort out of blankets or boxes, running very fast, or being able to whistle. The child sits on the chair and turns their head to look at the interviewer. “They run and jump. Run superfast. But do you know who is the best at running? It’s Peter.”

Most of the time, the children believe in their own abilities and that they can do everything their friends at preschool can do. They feel they have the same movement skills as one another, as they tend to copy each other and learn the same movement skills at preschool. The children also voice their self-confidence by expressing that they sometimes have more movement skills than their friends and that they have nothing else to learn from them, which is shown in the following:

- I: Is there anything your friends at preschool can do that you cannot?
- P: [Stands on the floor and starts walking around the room in big circles] “No, there is nothing.”
- (Participant 5)

### Physical competence

The category emphasises children’s physical competence, showcasing their ability to develop and use movement skills in various contexts and environments. They demonstrate these skills explicitly and implicitly through play, expressing themselves less through emotions and in a more matter-of-fact manner. The children engage in both free play and organised activities led by preschool teachers and instructors. The category includes two subcategories: *Having movement skills in free play* and *Taking part in organised play.*

#### Having movement skills in free play

The subcategory shows the physical competence that the children say they have, as well as the movement skills they implicitly express. Through play, the children highlight their abilities, and this takes place in different settings and environments, indicating the breadth of their competence.

The children describe specific movement skills when reflecting on what they can do when they move their bodies. They explicitly voice their competence in, for example, running, jumping, riding a bicycle, climbing on rocks and in trees, as well as dancing. The children also describe their skills in acrobatic movements such as cartwheels, handstands, and somersaults, as well as movements that require assistance from a friend, such as the wheelbarrow, which is shown in the following:

- I: What do you do when you move your body?
- P: “Ehh… I like to do the wheelbarrow. But you have to be two to do it. Because you do it like this, and someone else holds my legs.” [Puts both hands on the floor and lifts their left leg up and straight back, with the right foot still on the floor].
- I: Oh yes, are you good at it too?
- P: “Yes. Then you…” [Stands up and moves both hands in the air imitating crawling].
- (Participant 3)

The children play interactive games such as “the floor is lava” or chase. They highlight their physical competence through games where they adapt certain movements of a character they pretend to be. These games can be based on fictional cartoon characters, or they play, for example, can play, for example, with unicorns. The characters come with specific attributes that the children copy, thereby moving like them, implicitly expressing their movement skills in these games. The children also talk about what they play in preschool, which relates to their physical competence and fine motor skills, such as playing with cars and building with Lego or building blocks. They also draw, play with beads, and play a variety of different games with their friends, such as playing house and school, with toys and dolls, as well as pretending to be cats or dogs. However, the children do not see this as movement; they are simply playing, which is shown in the following:

- I: What do you usually do in the Lego room?
- P: [Sits on the chair, kicking both legs back and forth, looking straight ahead] “Build.”
- I: What do you build?
- P: [Looks at interviewer] “Not like a sandcastle, but a… tower.”
- I: Oh, fun.
- P: “And a house. Like a house that is taller than the ceiling.” [Points with their entire right arm straight up towards the ceiling with their index finger extended].
- (Participant 2)

Whilst talking about things they do at preschool, the children also highlight their physical competence in different settings, such as indoors, outdoors, in the playground, and in the woods. When playing in the playground at preschool, they like to use the swings, the slide, and the sandbox, where they build sandcastles, dig in the sand, and bury toys for their friends to dig up. The children explore the woods adjacent to the preschool, where they jump and climb on rocks and trees, as well as look for pinecones, sticks, and acorns, which they play with.

The children also describe their abilities to move in different environments, for example, when it is raining, in snow, on ice, and in warm weather. They adapt how they play depending on the conditions in these environments, describing their abilities to play, for example, in snow and water. During the winter, the children explain how they build snowmen and make high piles of snow. This indicates that they have movement skills in rolling snowballs, building, and climbing on snow. They also make snow angels and go sledging. When the children go sledging, they walk up the hill whilst pulling the sledge, and then go down, using their feet to brake. This highlights the children’s range of unvoiced movement skills. Their competence in different environments is shown in the following:

- I: What do you like to do outside?
- P: [Sits on the chair, with both hands under their legs, whilst looking at the interviewer] “When there’s snow, I like to make a big pile.”
- I: OK. What do you do with the snow pile?
- P: “I put snow in a bucket, and we put it, so it becomes a really big pile. Then we can jump in it.”
- I: That sounds like fun. Do you climb on it and jump?
- P: “Yes.”
- (Participant 1)

#### Taking part in organised play

In this subcategory, the children describe their movement skills in organised activities. The activities are organised by the teachers at preschool, both outdoors and indoors, and during leisure time by instructors in organised sports clubs. These activities exemplify the various opportunities for the children to practice and develop new physical competencies.

The children say that the preschool teachers decide when they are going outside and sometimes organise shared movement activities for the children whilst outside. The outdoor activities include, for example sledging in the winter, playing football, and doing scavenger hunts looking for pinecones and insects in the spring and autumn: *The child points to the yard*, sledging in the winter, playing football, and doing scavenger hunts looking for pinecones and insects in the spring and autumn. The child points to the yard. “We usually do obstacle courses. You have to have hoops, and then you have to jump in them. Then you have to jump count and jump around in the hoops.” These descriptions indicate that the children use a range of different movement skills in order to participate in the outdoor activities.

The children report that the teachers also organise movement activities indoors, though with somewhat more limited options compared to outdoors. During the movement activities, the children follow the movements demonstrated by the teachers. The activities can be, for example, in the form of a gymnastics session or a dance session where the children also dance freely to the music. The children also share that Santa’s elf gives them movement missions during the Christmas season. The children say that they enjoy receiving letters from the elf and following the instructions on how they should move on that specific day, thus developing their physical competence in the activity. This is shown below:

- I: Did you get a letter from the elf today?
- P: “We haven’t looked yet.”
- I: You have to do that later then.
- P: [Walks over to a shelf and picks up an old letter from the shelf] “Do you know what it says here? Today is my birthday, and how old are you? Jump back and forward the same number of times as your age. So, look, this is how many years old I am!” Jumps with both feet backwards five times. Then stops and jumps with both feet forward five times.
- (Participant 3)

Outside preschool, the children participate in leisure activities where they develop their movement skills further. They describe how they partake in organised activities led by instructors, for example, football, wrestling, riding, and parkour. Gymnastics is also an organised activity where the children describe learning, practising their physical competence by doing somersaults on a trampoline and walking on the balance beam.

## Discussion

A key insight emerging from the children’s interviews was that the four components of physical literacy (knowledge and understanding, motivation, self-confidence, and physical competence), appear to be closely integrated within its three domains (physical, cognitive, and affective) [19]. The cohesion among these components reflects the fundamental principles of physical literacy, which are grounded in a holistic view of the human being [18]. This finding reinforces the idea that the development of physical literacy depends on the presence of, and interaction between, all four components [29].

An important observation from the results is that the children perceived the relationship between their physical competence and self-confidence in movement as uncomplicated. Another key insight is that movement was experienced as emotionally meaningful for the children. They appear to associate movement with their feelings, and their engagement in movement was influenced by interactions with other children and adults in play and movement activities. The children voiced their knowledge, understanding, and experiences of movement based on how they felt, whether happy or uneasy, and how their bodies responded during the activity. This finding aligns with the characteristics of the affective domain, which encompasses motivation and enthusiasm for engaging in movement [19]. Furthermore, it adds to the existing body of knowledge on this domain, as children’s own perspectives on feelings are largely unexplored, despite their importance for the development of physical literacy [19, 30, 31]. The results also show that preschool children are motivated to move primarily for enjoyment, rather than because they are told to. This highlights the importance of promoting children’s physical literacy, rather than focusing solely on physical activity. As motivation for movement appears closely connected to feelings, children may be less inclined to move when coerced. By promoting the affective domain of physical literacy [19], children can develop a positive motivation for movement. Thereby, they can choose to be active without adults needing to persuade them. Throughout the interviews, the children continuously made micro-movements, such as playing with toys or moving around the room instead of sitting still, emphasising the natural and inherent aspect of movement. Children are not meant to be still, nor do they wish to be.

The children expressed enjoyment when engaging in play and movement activities with their friends and teachers, highlighting the social aspect of physical literacy as an important motivator for movement [29]. Through play and interaction, children can learn from and support each other in developing confidence in movement. Social interaction may also promote a sense of belonging and well-being, which are important for the development of physical literacy, especially within the affective and social domains [19, 29]. Importantly, these findings should partly be understood as context dependent. Through its organisational design, the Swedish preschool promotes social interaction [11], as children engage daily with peers of the same age and with teachers who actively participate in play alongside them. Such conditions may not be equally present outside the preschool setting, such as in the home environment, and may also differ across countries and cultural contexts. Consequently, the Swedish preschool may provide unique opportunities to support children’s motivation for movement and physical literacy development [32].

However, the children described situations in which teachers or peers decided on activities without involving them. This generated a feeling of exclusion and, consequently, reduced their motivation to move. Lindberg and Hedenborg emphasise that co-determination is essential for creating a safe and encouraging movement environment in which children can thrive and develop through movement [33]. Promoting children’s movement may therefore benefit from a stronger focus on co-determination. At the same time, Lindberg and Hedenborg argue that promoting co-determination requires both conscious awareness and intentional practice in order for it to become integrated into everyday preschool routines [33]. Batorowicz et al. [34], describe child development as shaped through dynamic interactions with the social environment, mediated by the child’s subjective experiences of people, places, materials, and cultural norms. Their model highlights three key mechanisms: choice, active engagement, and collaboration. These mechanisms were clearly reflected in the children’s narratives: they were more motivated to move when they could influence activities (choice), described movement in terms of emotions and bodily impulses (active engagement), and expressed enjoyment when engaging in movement activities with others (collaboration).

This indicates that movement is socially constructed and context-dependent, aligning with both Batorowicz’s model and Whitehead’s theory of physical literacy [19, 34]. Whitehead further argues that movement is an embodied experience, intrinsically tied to both identity and emotion [18, 19]. The children’s reflections on how movement made them feel, and how they sensed and listened to their own bodies, demonstrate that even at a young age they relate to their bodily selves in intuitive and meaningful ways. The concept of embodiment thus adds a crucial layer to understanding children’s perspectives on movement. The children experienced movement in relation to their feelings and surroundings, illustrating the close connection between embodiment and emotion. This connection is further emphasised through the role of embodied self-expression in communicating attitudes, moods, and personality, underscoring that physical capacities are central to emotional and interpersonal expression [18].

Furthermore, adopting an embodiment perspective may strengthen the rationale for exploring physical literacy through qualitative methods. To understand and support the development of physical literacy in early childhood, it is imperative to consider how children *experience* and *interpret* movement, not only what they are able to *perform*. This perspective is essential when designing interventions that aim to promote physical literacy in ways that are meaningful, inclusive, and sustainable.

### Limitations and strengths

Some limitations should be acknowledged. The study was restricted to preschools whose principals consented to data collection, and it proved difficult to gain access to preschools in socioeconomically disadvantaged areas. This may have led to limited variation in the backgrounds and prerequisites of the participating children. Furthermore, since legal guardians gave consent prior to the children doing so, those with a personal interest in movement may have been more likely to encourage their child’s participation. This in turn may have contributed to a less diverse representation of children’s perspectives. Future research should therefore make efforts to include preschools from a broader range of socioeconomic contexts.

During the interviews, the children were asked if they wanted to demonstrate their movements outdoors in the preschool yard, which only four children chose to do. However, as all interviews were video-recorded, children’s movements were nonetheless captured indoors, providing valuable complementary data. This approach enabled a deeper understanding of children’s physical literacy than would have been possible through interviews alone.

The interaction during the interviews may have been affected by the power structure inherent in adult-child relationships. However, the study was designed to minimise the effects. For example, the first author spent time at the preschools prior to the interviews to help the children feel more comfortable. The interviews were carried out in a familiar environment to support relaxation, and emphasis was placed on following the children’s needs during the interviews. Two children chose not to participate and appeared comfortable communicating their withdrawal of consent to the first author. The other children displayed a clear desire to participate, showing interest in the interviews beforehand and eagerly asking if they could be interviewed next.

A key strength of this study is its added value in addressing a gap in the physical literacy literature by foregrounding children’s own perspectives. Specifically, the findings provide insight into how children experience, interpret, and make sense of movement and physical literacy in everyday preschool life, rather than primarily capturing known outcomes such as the amount of movement (e.g., step counts or minutes of physical activity) or motor skills performance [35–37]. In addition, by situating children’s perspectives within the Swedish preschool context, the study offers a nuanced understanding of how contextual conditions may enable or hinder children’s movement and engagement. Taken together, these contributions provide important insights into how to promote meaningful movement experiences and support the development of a lifelong relationship with movement.

Although children’s perspectives on physical literacy were the focus of this study, it is important to acknowledge preschool teachers as a key contextual factor in the preschool setting. The teachers’ perspectives could deepen the understanding, as they create movement opportunities and can highlight contextual conditions that influence the children’s physical literacy. Further research that includes teachers’ perspectives, as well as their prerequisites for supporting children’s movement, would therefore be of interest.

## Conclusions

This study contributes to the scarce body of research on preschool children’s physical literacy. The findings highlight that children’s motivation for movement was closely connected to their emotions, thereby recognising the affective domain as important for movement from the children’s perspectives. Yet, from a physical literacy perspective, the integration of the affective, physical, and cognitive domains is essential for holistic development in early childhood. By incorporating children’s own perspectives, this study advances current understandings of physical literacy and emphasises the value of considering children’s experiences in both research and practice. These insights should be taken into account in future studies and in efforts to promote physical literacy in preschool settings.

## Supplementary Information

Supplementary Material 1.

## Data Availability

Data generated and analysed in this study are not publicly available due to ethical restrictions and to ensure the confidentiality of the informants.
