# Supplementary material for: “I just want to move": preschool children’s perspectives on their physical literacy
Source: BMC Public Health. 2026 Jan 27;26:690. doi: 10.1186/s12889-026-26403-7 (PMC12924250; doi:10.1186/s12889-026-26403-7)
Supplement: Supplementary file 1 — Supplementary Material 1. [file 12889_2026_26403_MOESM1_ESM.pdf]

Interview Guide- Preschool Children

1. **Competence: Own Physical Ability During Play**

   Tell me what kinds of body movements you can do

   (What can you do with your body, how can you move?)

   Which movement do you think you are best at? Why are you best at that one?

   Is there something you cannot do that you would like to be able to do?

   What makes you unable to do it?

2. **Motivation and Self-Confidence**

   Which movement is your favourite/the most fun? Why is it the most fun?

   What do you think is not fun to do? Why is it fun?

   Which movement would you like to try?

   If you could decide- which movement would you like to be able to do?

   (What would you like to be able to do? (climb, jump, run fast?))

   Do you want to learn new movements?

   Do you dare to try new movements? (What do you do to feel brave?)

   If you think about what your friends can do- is there something you would like to be able to do? (What and why?)

   Is there any movement you think is difficult? Why is it difficult?

   What do you do if something is a little hard?

   Do you like to move? (Why/Why not?)

   Do you want to move? (Why/Why not?)

3. **Participation and Engagement**

   Tell me what you do when you play at preschool

   Do you get to be part of decisions of what you play at preschool?

   Would you like to be able to decide?

   If you could decide, what would you play or what movements would you do then?

   Would you like to move if you could choose which movements you would do?

4. **Knowledge and Understanding (Health)**

   What happens in your body when you play and move?

How does it feel in your body when you play and move?

Do you know what is good for your body?

Why is it good for your body?

How does it feel in your body when it feels good?

Where in your body do you feel it?

How does your body feel when you sit still for a long time?

Why is it important to move?
